# Kidney-inspired algorithm with reduced functionality treatment for classification and time series prediction

**DOI:** 10.1371/journal.pone.0208308

**Published:** 2019-01-04

**Authors:** Najmeh Sadat Jaddi, Salwani Abdullah

**Affiliations:** Data Mining and Optimization Research Group (DMO), Centre for Artificial Intelligence Technology, Faculty of Information Science and Technology, National University of Malaysia, Bangi, Selangor, Malaysia; Liverpool John Moores University, UNITED KINGDOM

## Abstract

Optimization of an artificial neural network model through the use of optimization algorithms is the common method employed to search for an optimum solution for a broad variety of real-world problems. One such optimization algorithm is the kidney-inspired algorithm (KA) which has recently been proposed in the literature. The algorithm mimics the four processes performed by the kidneys: filtration, reabsorption, secretion, and excretion. However, a human with reduced kidney function needs to undergo additional treatment to improve kidney performance. In the medical field, the glomerular filtration rate (GFR) test is used to check the health of kidneys. The test estimates the amount of blood that passes through the glomeruli each minute. In this paper, we mimic this kidney function test and the GFR result is used to select a suitable step to add to the basic KA process. This novel imitation is designed for both minimization and maximization problems. In the proposed method, depends on GFR test result which is less than 15 or falls between 15 and 60 or is more than 60 a particular action is performed. These additional processes are applied as required with the aim of improving exploration of the search space and increasing the likelihood of the KA finding the optimum solution. The proposed method is tested on test functions and its results are compared with those of the basic KA. Its performance on benchmark classification and time series prediction problems is also examined and compared with that of other available methods in the literature. In addition, the proposed method is applied to a real-world water quality prediction problem. The statistical analysis of all these applications showed that the proposed method had a ability to improve the optimization outcome.

## Introduction

Nowadays, artificial neural networks (ANNs) are learning models that are commonly used to find functions that are unknown. A wide variety of optimization algorithms have been used to train ANNs [1–5]. Among these, the genetic algorithm (GA) is the most commonly used (e.g., [5–7]. However, several other algorithms have also been used to train ANNs [3, 8–15]. There are also enhanced versions of many of the earlier optimization methods in the literature [16–22].

This paper presents an enhancement of the kidney-inspired algorithm (KA) proposed in [23]. The KA, which is inspired by urine formation in the kidneys of the human body, starts with an initial population of units of water and solutes (solutions). The base of the KA is filtration of blood. This filtration is performed according to a filtration rate (*fr*). The *fr* in the basic KA is a mean objective function in the population. In the KA, the most promising solutes are transferred to filtered blood (FB) and the rest are moved into waste (W). In addition, reabsorption, secretion, and excretion are performed based on some condition during the search process. The best solution and the *fr* are updated in each iteration. At the end of each iteration FB and W are merged. The KA has already been used in many applications in the literature [24–26].

In the medical field, the health of the kidneys is measured by the glomerular filtration rate (GFR), which ranges between 0 and 120 [27]. In the human body, kidney function is considered normal if the GFR is greater than 60. If the GFR is between 15 and 60, kidney disease is present and the patient has to undergo treatment to improve the functionality of the kidneys. This treatment can be in form of taking medicine and/or changing dietary habits as recommended by medical doctors. If the GFR is less than 15, it means that kidney failure has occurred and the blood must be filtered by using a dialysis machine that acts in place of the patient’s kidney(s).

The enhancement of the KA presented in this paper is a simulation of the treatment employed to counteract the reduced functionality of the kidneys in the human body, in which the GFR test is used to check kidney function and to identify a suitable simulation of one of the real-world treatments mentioned above, and add it to the basic KA. This novel simulation is as following process: The GFR testing process is added to the KA after meeting all solutes in the population at the end of each iteration. The GFR level is calculated based on the *fr* of the current population and the *fr* of the FB population. The proposed method is designed to address both minimization and maximization problems, therefore two separate formulas for GFR are given. If the GFR level is less than 15, the KA process is repeated with the population in FB. This process is a simulation of the dialysis that is administered for kidney failure in the real world. If the GFR level is between 15 and 60, a movement of virtual solutes in FB is applied as a treatment for reduced kidney function. This movement increases the KA’s exploration capability and is intended to assist the algorithm in finding a better solution. If the GFR level is greater than 60, this denotes that kidney function is normal, in which case no additional process is added to the KA (i.e., a basic KA is used).

The rest of this paper is organized as follows: Section 2 provides a brief explanation of the KA. Section 3 describes in detail the simulation of the treatment for reduced functionality of the KA and how it is embedded in the basic KA. Section 4 discusses the experimental results of applying the proposed method to a number of test functions, benchmark classification and time series prediction datasets, and real-world water quality data. Finally, Section 5 presents a summary of the work and some conclusions.

## Kidney-inspired algorithm

The KA is an optimization algorithm that was recently proposed in [23]. The KA is inspired by the role of the kidneys in urine formation and blood filtration in the human body. Essentially, the kidneys manage the amount of ions in the blood and also reduce the presence of surplus water and waste. The KA mimics the four processes performed by the kidneys: filtration, reabsorption, secretion, and excretion. The process of filtration in the kidneys begins in the glomerular capillaries through which dissolved matter is transferred into tubules and then reabsorption is carried out. The solutes are returned from the tubules to the bloodstream by a process of reabsorption. The transfer of solutes to the renal tubules is considered as secretion. The solutes in renal tubules can be excreted in the urine.

In the initialization step of the KA, a random population of solutions is created. Then, the objective functions of all the solutions are calculated (according to the problem at hand). Eq (1) represents the movement of the virtual solutions in the KA:

$$Sol_{i+1}=Sol_{i}+rand\;(Sol_{best}-Sol_{i})$$ (1)

where *Sol* is a solution in the population of the KA, *Sol*_*i*_ is a solution in the *i*^*th*^ iteration, *rand* is a random number, and *Sol*_*best*_ is the best solution found so far.

**Fig 1:**
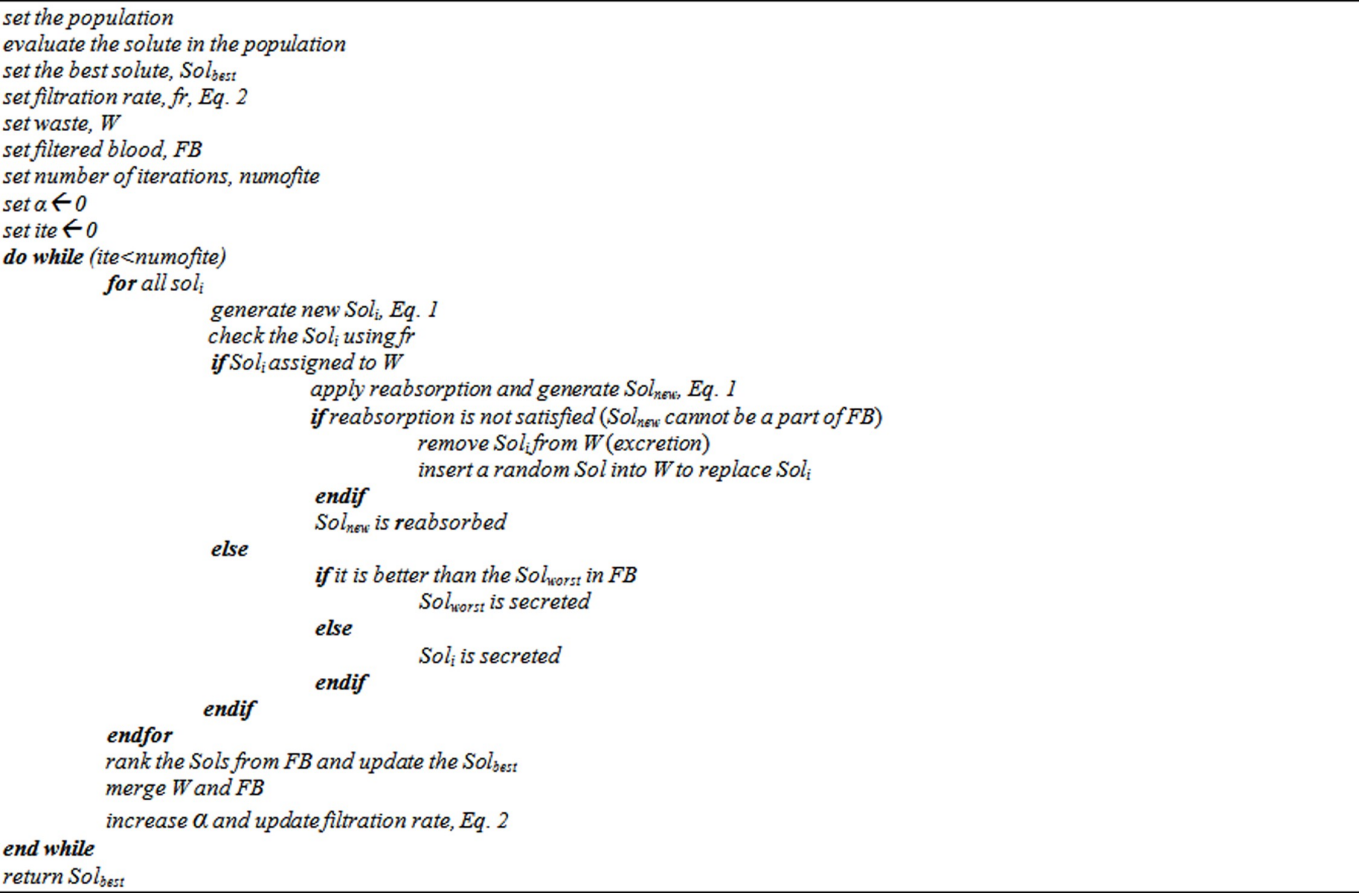
Pseudocode of KA.

The solutions with higher quality are filtered into FB and the rest are moved into W. The filtration rate, *fr*, is computed as follows:

$$fr=\alpha \times \frac{\sum_{i=1}^{p}f(x_{i})}{p}$$ (2)

where α is a constant value between 0 and 1, *p* is the population size, and *f*(*x*_*i*_) denotes the objective function of solution *x* in the *i*^*th*^ iteration. The pseudocode of the KA is shown in Fig 1 below:

The algorithm follows the rule below to decide whether to accept the solution as a member of FB or W (this is the filtration operator):

- If the quality of the solution is better than *fr*, accept the solution as a member of FB.
- If the quality of the solution is not better than *fr*, accept the solution as a member of W.

If a solution is a member of W, it has a chance to move to FB if the criterion for inclusion in FB is satisfied. Otherwise, excretion of the solution is carried out, and a random solution is inserted into W. In contrast, if after applying the filtration operator, a solution is a member of FB and its quality is not better than the worst solution in FB, it is secreted from FB. While, if the solution is better than the worst, the worst solution is secreted. Then the best solution is updated, after which FB and W are merged and the *fr* is updated. The process is repeated until a termination criterion (maximum number of iterations) is met. A full explanation of the KA can be found in [23].

Finding the global optimum is the main objective in all optimization algorithms. This aim is achieved by a trade-off between exploration and exploitation in any optimization algorithm. In KA, exploration is provided by the movement of solutes and process of filtration, whilst reabsorption provides the algorithm with exploitation. There is an open issue for enhancement of a balance between exploration and exploitation in order to improve the performance of the algorithm. The proposed method in this paper follows the contribution towards this enhancement.

## Treatment for reduced kidney function in the KA

In the human body, kidney disease occurs when one or both kidneys become damaged and can no longer function properly. When this happens, the patient needs to undergo some treatment in order to delay or stop further deterioration of the kidneys. The type of treatment employed depends on the severity of the disease. The three main treatment modalities are lifestyle changes, medication, and dialysis [27]. As kidney disease is progressive, it may lead to kidney failure, that is to say, the kidneys stop functioning entirely. When this occurs, a dialysis machine is used to replicate the functions of the kidneys. The dialysis machine filters the blood outside the body.

**Fig 2:**
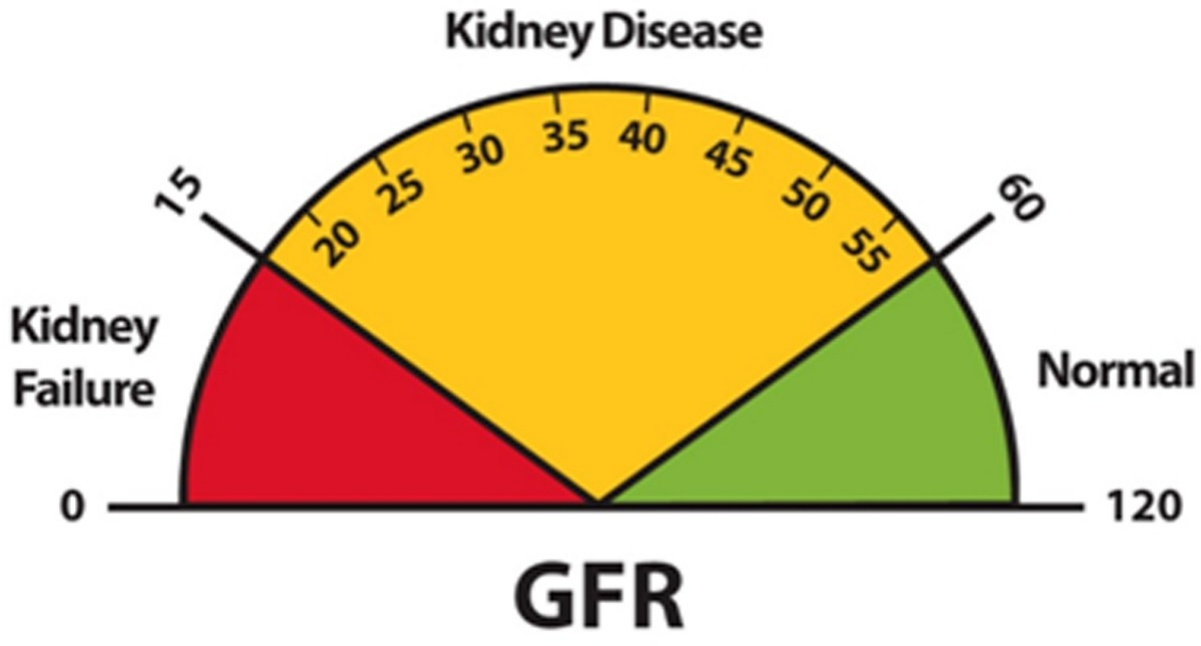
Kidney function chart [27].

A GFR test is performed to determine the presence and extent of kidney disease in the human body. The GFR is a measure of how well the kidneys are filtering blood and its value falls between 0 and 120. A GFR of 60 or higher is in the normal range and no treatment is needed. A GFR between 15 and 60 means kidney disease is present and some medication and/or other treatment is needed. A GFR of 15 or lower may be indicative of kidney failure [27], where greater intervention in the form of dialysis is required. A chart that illustrates the levels of kidney function is shown in Fig 2.

The above conceptualization acts as the inspiration for the additional simulations for the KA that are presented in this paper. The details of the KA with treatment (coded as KAT) are described in the following subsections.

### Calculation of the GFR level

In each iteration of the KA, after meeting all solutes and before merging the FB and W as the population for the next iteration, the algorithm calculates the GFR level based on the *fr* in FB. The GFR is calculated by Eq 3 for minimization problems and by Eq 4 for maximization problems:

$$GFR_{\min}=120-(\frac{fr_{FB}^{}*100}{fr})$$ (3)

$$GFR_{\max}=\frac{fr_{FB}*12}{fr}$$ (4)

where in both Eqs (3) and (4) *fr* is the filtration rate for the current population and *fr*_*FB*_ is the filtration rate for the current population in FB which is the average of objective functions in the current FB. The GFR for both minimization and maximization problems can fall between 0 and 120.

### GFR level greater than 60

If the GFR is greater than 60, the algorithm is performing the filtration process well and there is no need to apply any treatment. In other words, high-quality solutions are being filtered and moved into FB and the algorithm can continue the same process as the basic KA.

### GFR level between 15 and 60

If the GFR is between 15 and 60, the algorithm needs some treatment to improve the filtration operation. This happens if the quality of the solutions in FB is not very good. The treatment involves the movement of virtual solutions in FB by using Eq 1. This formula is applied for all solutions in FB to cause more exploration and this may help the algorithm to find better solutions. This is a simulation of changing the lifestyle or medication of a patient in the real-world in order to achieve better kidney function.

### GFR level less than 15

**Fig 3:**
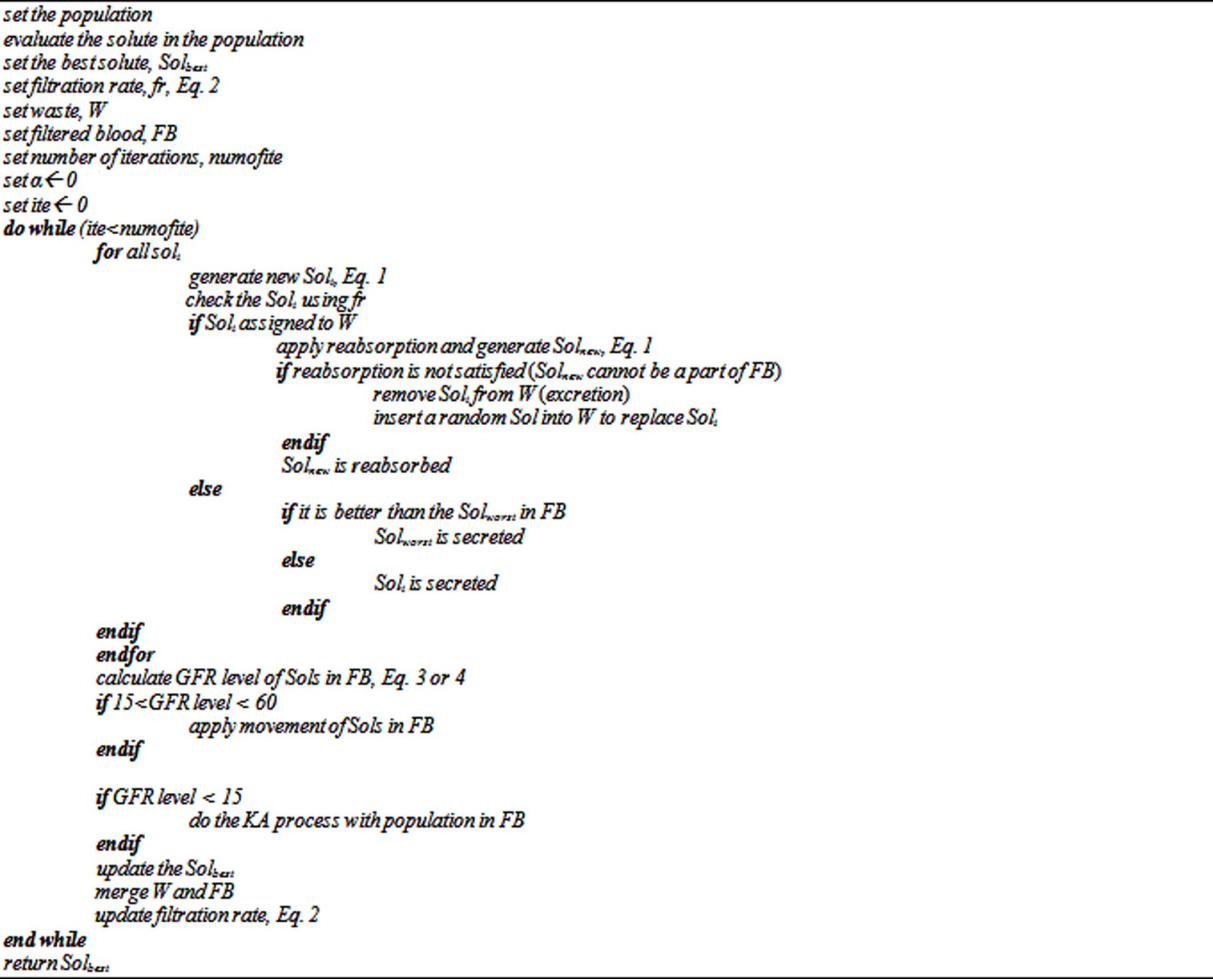
Pseudocode of KAT.

If the GFR is less than 15, the filtration operation in the algorithm is not functioning at all. This happens when the solutions in FB are really poor. To overcome this issue a repetition of the KA process is performed on a FB population. This is a simulation of repeating the blood filtration process by dialysis machine in the real world. With this simulation, the processes of filtration, reabsorption, secretion and excretion are performed with the FB population and this facilitates more exploration so that the algorithm may be able to find a better solution. The pseudocode of the KAT is shown in Fig 3 and the flowchart of KAT is presented in S1 Fig.

## Experimental results

In order to test and evaluate the proposed method (KAT), first it is applied to eight well-known test functions and its results are compared with those of the KA. Second, the performance of the KAT on benchmark classification and time series prediction problems is examined and compared with that of the KA. The classification and time series prediction results of the KAT are also compared with those of other methods in the literature. Finally, the KAT is applied to real-world water quality prediction, the results of which are discussed in final part of this subsection.

### Results for test functions

The KAT and the KA are applied to eight test functions. The number of iterations is set to 100 and the number of solutions in the population (population size) is also set to 100. These values are chosen because they have been used in the related literature [28–30]. The details of the test functions that were used to test the performance of the KAT and compare it to that of the KA are given in Table 1. Note that the value of *d* in parenthesis is a constant value which is set in the test function formula.

**Table 1:** Details of the test functions.

Test function	Equation for test function	Global optimum	Dimension
Michalewicz(*d* = 2)	$f(x)=-\sum_{i=1}^{d}\sin(x_{i})\sin^{2m}\left(\frac{ix_{i}^{2}}{\pi }\right)$	*f* (*x*) = -1.8013*x* = (2.20, 1.57)	0 < *x* < π
Rosenbrock(*d* = 16)	$f(x)=\sum_{i=1}^{d-1}\left[100{\left(x_{i+1}-x_{i}^{2}\right)}^{2}+{\left(x_{i}-1\right)}^{2}\right]$	*f* (*x*) = 0*x* = (1, 1)	-2.048 < *x* < 2.048
De Jong(*d* = 256)	$f(x)=\sum_{i=1}^{d}x_{i}^{2}$	*f* (*x*) = 0*x* = (0, 0)	-5.12 < *x* < 5.12
Schwefel(*d* = 128)	$f(x)=418.9829d-\sum_{i=1}^{d}x_{i}\sin(\sqrt{\left|x_{i}\right|})$	*f* (*x*) = 0*x* = (420.9687, 420.9687)	-500 < *x* < 500
Ackley (*d* = 128)	$f(x)=-20\exp(-0.2\sqrt{\frac{1}{d}\sum_{i=1}^{d}x_{i}^{2}})-\exp(\frac{1}{d}\sum_{i=1}^{d}\cos(2\pi x_{i}))+20+e$	*f* (*x*) = 0*x* = (0, 0)	-32.768 < *x* < 32.768
Rastrigin	$f(x)=10d+\sum_{i=1}^{d}\left[x_{i}^{2}-10\cos(2\pi x_{i})\right]$	*f* (*x*) = 0*x* = (0, 0)	-5.12 < *x* < 5.12
Easom	$f(x)=-\cos(x_{1})\cos(x_{2})\exp(-{\left(x_{1}-\pi \right)}^{2}-{\left(x_{2}-\pi \right)}^{2})$	*f* (*x*) = -1*x* = (π, π)	-100 < *x* <100
Griewangk	$f(x)=\sum_{i=1}^{d}\frac{x_{i}^{2}}{4000}-\prod_{i=1}^{d}\cos\left(\frac{x_{i}}{\sqrt{i}}\right)+1$	*f* (*x*) = 0*x* = (0, 0)	-600 < *x* < 600

The results for the eight test functions are provided in Table 2. The algorithm was run 100 times. The average and the best result achieved by the KAT as well as by the KA for each test function are reported in this table.

**Table 2:** Results for the test functions.

Test function	Optimum	KA		KAT	
		Mean	STD	Mean	STD
Michalewicz	-1.8013	-1.8142	3.20E-04	-1.8013	0
Rosenbrock	0	0	0	0	0
De Jong	0	0	0	0	0
Schwefel	0	2.00E-05	2.30E-06	0	0
Ackley	0	0	0	0	0
Rastrigin	0	0	0	1.23E-08	3.72E-09
Easom	-1	-1	0	-1	0
Griewangk	0	0	0	0	0

As can be seen from Table 2, the KAT achieves the global best in all 100 runs for seven out of the eight test functions. In comparison with the KA, the KA cannot meet global best in all 100 runs in two test function. The superiority of the performance of the KAT is due to the treatment that is applied when the algorithm is working with reduced functionality. This treatment enhances the exploration capability of the algorithm because when the GFR is less than 15, the four processes–filtration, reabsorption, secretion and excretion–are performed again for solutions in FB and therefore more exploration capability is provided for the algorithm.

In order to compare the KAT with the KA, the number of function evaluations the algorithms need to make is computed for when the algorithms achieved the global optimum. The average of the number of function evaluations for 100 results is reported in Table 3. As can be seen, the KAT has to make more function evaluations compared to the KA in all cases. This is due to the objective function recalculation that takes place during the treatment in the KAT. While the treatment improves the performance of the algorithm a greater number of function evaluations need to be carried out to find the better solution.

**Table 3:** Comparison of number of function evaluations made by KA and KAT.

Test function	KA		KAT	
	Mean	STD	Mean	STD
Michalewicz	**3301**	428	3581	243
Rosenbrock	**5663**	1283	6532	1039
De Jong	**5829**	542	6289	628
Schwefel	**8810**	623	9525	432
Ackley	**4736**	769	5172	714
Rastrigin	**9785**	3211	9861	5261
Easom	**5673**	892	5948	725
Griewangk	**8239**	3846	9250	4915

**Fig 4:**
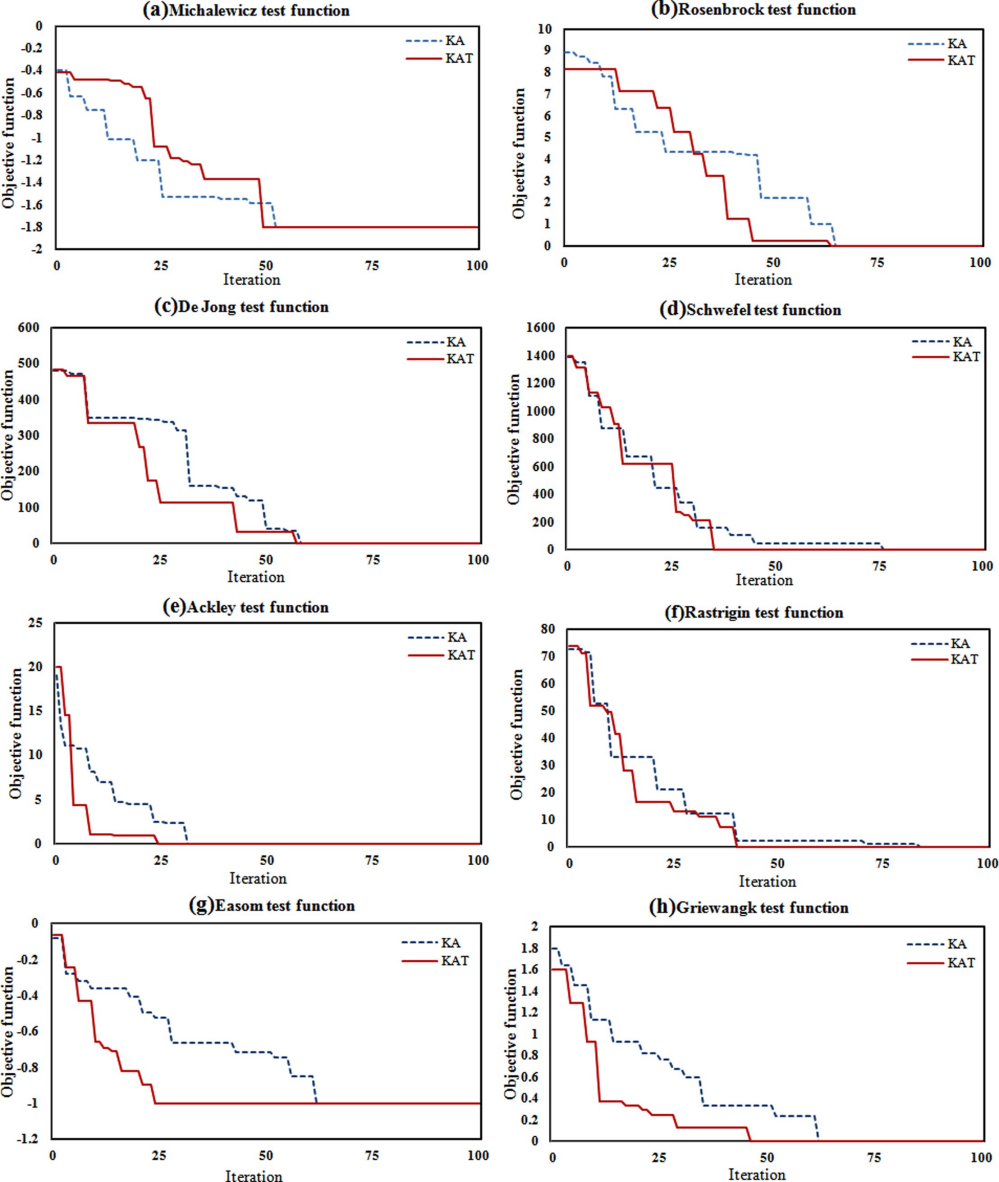
Optimization progress of KA and KAT for: (a) Michalewicz, (b) Rosenbrock, (c) De Jong, (d) Schwefel, (e) Ackley, (f) Rastrigin, (g) Easom and (h) Griewangk test functions.

The optimization progress of the KA and KAT for the eight test functions is illustrated in Fig 4. From a comparison of the convergence in the KA and KAT, it can be seen that the KAT can find the optimum solution earlier than the KA most of the time. However, the results in Table 3 show that the KAT has more function evaluations (due to the treatment process) compared to the KA. So it can be concluded that although the KAT makes more calculations of the objective functions in each iteration (based on Table 3), it is able to achieve the best results in earlier iterations, which compensates somewhat for the time taken to perform the objective function evaluation in the treatment process.

### Results for benchmark classification and time series prediction problems

The performance of the KAT on classification and time series prediction problems is also examined and compared with that of the KA. The details of the datasets used in this evaluation are presented in Table 4. The first six datasets in this table are benchmark classification datasets and the last two are benchmark time series prediction datasets.

**Table 4:** Characteristics of datasets.

Dataset	No of examples	No of features	No of classes
Iris (https://archive.ics.uci.edu/ml/datasets/iris)	150	4	3
Diabetes (https://archive.ics.uci.edu/ml/datasets/diabetes)	768	8	2
Thyroid (https://archive.ics.uci.edu/ml/datasets/thyroid)	7,200	21	3
Cancer (https://archive.ics.uci.edu/ml/datasets/Breast+Cancer+Wisconsin+(Diagnostic)))	699	10	2
Card (https://archive.ics.uci.edu/ml/datasets/default+of+credit+card+clients)	690	15	2
Glass (https://archive.ics.uci.edu/ml/datasets/glass+identification)	214	10	6
Mackey-Glass (https://figshare.com/articles/Mackey-Glass_time_series/4233584/1)	1,000	1	0
Gas Furnace (http://datasets.connectmv.com/datasets)	296	2	0

The UCI machine learning repository [31] is the source of the classification datasets (the first six datasets). The Mackey-Glass time series dataset is calculated from an equation taken from the literature [11].The Gas Furnace time series dataset can be found at http://datasets.connectmv.com/datasets.

An ANN with two hidden layers with two nodes for each hidden layer is used for this experiment because previous studies have found that this structure is the best structure for this type of investigation [1, 12, 32]. The activation function for this experiment is the hyperbolic tangent because it presents better results compared to other options [33]. A one-dimensional vector with the weights and biases placed in each cell of the vector is employed for the solution representation. Any classifier suffers from curse of dimensionality. Therefore, feature selection is important as a pre-processing task for a classification problem [33–38[34–39]. Other methods in the literature have used the full feature set to test the performance of their method. Thus, in our work, the full feature set is also used in order to carry a fair comparison between our method and other available methods in the literature. For the classification problems, the inputs are conditional attributes and the output is the class attribute. For time series prediction, the output for the Mackey-Glass dataset is *x*(*t*+6) and the inputs are *x*(*t*), *x*(*t*-6), *x*(*t*-12), and *x*(*t*-18) and for the Gas Furnace dataset the output is *y*(*t*) and the inputs are *u*(*t*-3), *u*(*t*-2), *u*(*t*-1), *y*(*t*-3), *y*(*t*-2), and *y*(*t*-1). The inputs and outputs are set as in earlier works [1, 12]. In line with earlier studies [11, 32] 30 twofold iterations are employed to evaluate the results of the models. The data are normalized into the range of [−1, 1]. First, the performance of the KAT on classification and time series prediction problems is compared with that of the KA and then the KAT is compared with the methods in the literature.

#### Results of comparison of KA with KAT for classification and time series prediction problems

The results obtained by the KA and KAT when applied to some benchmark classification and time series prediction problems are presented in Table 5. For the classification problems the testing and training error is represented by the classification error, while for the time series prediction problems the root mean square error is employed for the Mackey-Glass dataset and the mean square error is used for the Gas Furnace dataset.

**Table 5:** Results for classification and time series prediction by KA and KAT.

Dataset	Criteria	KA	KAT
Iris	Training error %	2.4213	1.6377
	Std. Dev.	0.0547	0.0342
	Testing error %	2.6738	1.6575
	Std. Dev.	0.0726	0.0837
Diabetes	Training error %	19.1576	13.3303
	Std. Dev.	0.0028	0.0092
	Testing error%	20.8243	15.3251
	Std. Dev.	0.0625	0.0425
Thyroid	Training error %	6.7401	4.8717
	Std. Dev.	0.0172	0.0273
	Testing error%	7.6212	5.1436
	Std. Dev.	0.0726	0.0782
Cancer	Training error %	2.9947	2.8158
	Std. Dev.	0.0762	0.0629
	Testing error%	3.2514	2.8996
	Std. Dev.	0.0289	0.0417
Card	Training error %	14.6352	12.72
	Std. Dev.	0.0459	0.0276
	Testing error%	16.4859	13.0705
	Std. Dev.	0.0027	0.00376
Glass	Training error %	39.7632	30.0585
	Std. Dev.	0.0043	0.0065
	Testing error%	40.4299	31.6766
	Std. Dev.	0.0836	0.0982
Mackey-Glass	Training error %	3.20E-03	1.50E-03
	Std. Dev.	0.0001	0.0002
	Testing error%	3.47E-03	1.70E-03
	Std. Dev.	4.60E-06	0.000005
Gas Furnace	Training error %	0.3941	0.2383
	Std. Dev.	5.00E-06	7.00E-06
	Testing error%	0.4426	0.2664
	Std. Dev.	0.0072	0.0048

It can be seen from Table 5 that the KAT performs better than the KA in most of the cases. This is due to the treatment process embedded in the KAT which provides the algorithm with more exploration, which enables it to find the optimum solution with fewer training and testing errors.

**Fig 5:**
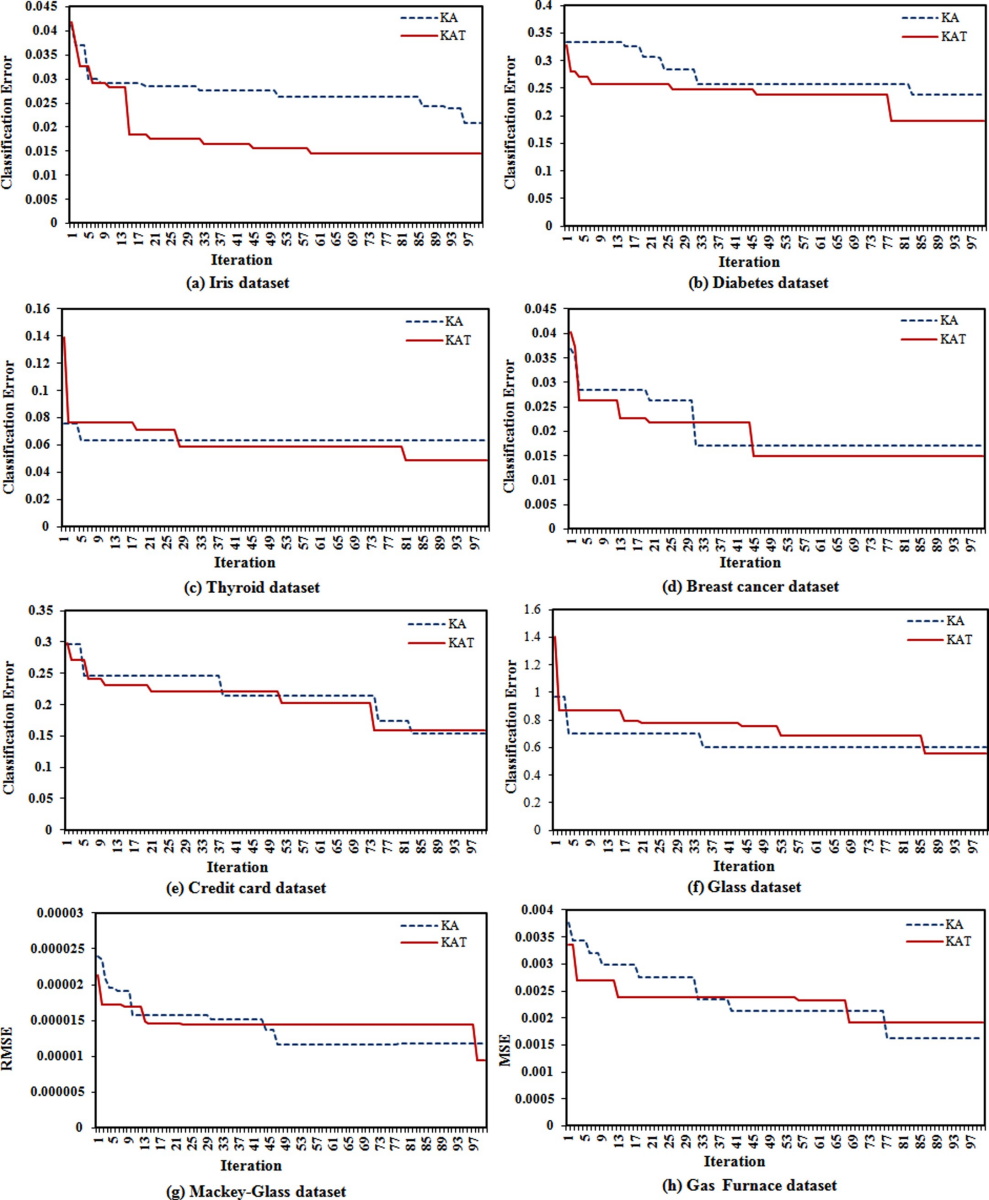
Comparison of optimization progress of KA and KAT in: (a) Iris dataset, (b) Diabetes dataset, (c) Thyroid dataset, (d) Breast cancer dataset, (e) Credit card dataset, (f) Glass dataset, (g) Mackey- Glass dataset, and (h) Gas Furnace dataset.

Fig 5 shows a comparison of the optimization progress of the KA and KAT over 100 iterations. From the graphs presented in the figure it can be observed that the KAT achieved the optimum solution in earlier iterations in most of the cases compared to the KA. This is due to the treatment process that was developed for the proposed algorithm. Although the treatment process is more time consuming (see Table 3), it can still lead the KAT towards better results in earlier iterations. There is better convergence in the KAT because more exploration is achieved by reabsorption and secretion during the treatment process of the KAT.

In order to determine whether the KAT is statistically different from KA, the *p-*values for all the datasets are calculated. The results are provided in Table 6. The critical value α is equal to 0.05. This statistical analysis is performed for both the training error and testing error. Values that are lower than the critical value are shown in bold. The results in Table 6 confirm that the KAT has better ability than the basic KA because the former’s *p*-values are much lower than the critical value and this supports the finding that the KAT outperforms the KA.

**Table 6:** Pairwise comparison of *p*-values.

Dataset	KA	
	Training error	Testing error
Iris	**2.59E-05**	**0.0006121**
Diabetes	**0.000188**	**5.153E-05**
Thyroid	**0.00922**	**6.629E-06**
Cancer	0.1873316	**0.0074388**
Card	**0.0133355**	**0.0009458**
Glass	**0.0006114**	**9.904E-05**
Mackey-Glass	**8.933E-06**	**8.029E-06**
Gas Furnace	**0.0001974**	**3.854E-05**

#### Comparison of the KAT with methods in the literature

A comparison of the performance of the KAT with that of methods in the literature when applied to benchmark classification and time series prediction problems is presented in Tables 7 and 8, respectively. For the classification problems, the performance is presented in terms of classification error and for the time series prediction problems it is represented by the time series prediction error. The best results are denoted in bold.

**Table 7:** Comparison of KAT and other methods for classification problems.

Dataset	KAT	BatRM-S	T-LogisticBatDNN	SA	TS	GA	TSa	GaTSa+BP
Iris	1.6575	**1.5309**	1.8313	12.649	12.478	2.5641	4.6154	5.2564
Diabetes	**15.3251**	19.3021	20.139	27.156	27.404	25.994	25.876	27.061
Thyroid	**5.1436**	6.2435	6.7545	7.3813	7.3406	7.285	7.3322	7.1509
Cancer	**2.8996**	2.9928	2.8947	7.1729	7.2779	7.422	6.2846	7.192
Card	**13.0705**	13.4163	13.738	23.469	18.042	31.724	21.269	15.242
Glass	**31.6766**	38.4251	38.429	58.381	56.412	58.031	57.777	55.142

**Table 8:** Comparison of KAT and other methods for time series prediction problems.

Dataset	KAT	BatRM-S	T-Logistic BatDNN	gHFSNN (triangular)	gHFSPNN (Gaussian)	gFSPNNT	gFSPNNT
Mackey-Glass	**1.70E-03**	0.0019	0.0027	0.0315	0.0262	0.118	0.0441
Gas Furnace	**0.2664**	0.5518	0.33	11.52	10.25	11.2	10.3

Table 7 shows the results of a comparison of the KAT and (1) a multi-population bat algorithm (BA) (BatRM-S) [11], (2) a BA with logistic map and parameter setting using the Taguchi method (T-LogisticBatDNN) [12], (3) simulated annealing (SA), (4) tabu search (TS), (5) a GA, (6) a combination of TS and SA (TSa), and (7) an integration of TS, SA, and GA with backpropagation (GaTSa+BP), which are all available in [32]. From the table it can be seen that the KAT outperforms four out of the seven methods. Table 8 shows the results of a comparison of the KAT with five methods that can be found in the literature [1, 12, 40]. From the table it is clear that the KAT achieved better results for both datasets, which confirms the ability of the KAT. The superior results of the KAT in both classification and time series prediction are due to the treatment process that is applied when the functionality of the KA is reduced. This provides the algorithm with more exploration in the search process and therefore leads the algorithm to superior solutions.

The Friedman test and Nemenyi post-hoc test are performed in order to determine whether there are significant differences between the methods in terms of classification and time series prediction errors.The Friedman test result for classification problems is 7.39444. This value is bigger than the critical value (5.99), so there is a significant difference in the performance of the tested algorithms. The results of the Nemenyi test are shown in Table 9, where the MSD is equal to 6.4807. It can be seen from the bolded figures in the table that there is a statistically significant difference in 13 cases.

**Table 9:** Nemenyi test for the classification problem.

Algorithms		KAT	BatRM-S	T-LogisticBatDNN	SA	TS	GA	TSa	GaTSa+BP
	Mean	11.62882	13.65178	13.96442	22.70153	21.49242	22.17002	20.5257	19.50738
KAT	11.62882	-	2.022967	2.3356	**11.07272**	**9.8636**	**10.5412**	**15.4743**	**7.878567**
BatRM-S	13.65178	-	-	0.312633	**9.04975**	**7.840633**	**8.518233**	**6.873917**	5.8556
T-LogisticBatDNN	13.96442	-	-	-	**8.737117**	**7.528**	**8.2056**	**6.561283**	5.542967
SA	22.70153	-	-	-	-	1.209117	0.531517	2.175833	3.19415
TS	21.49242	-	-	-	-	-	0.6776	0.966717	1.985033
GA	22.17002	-	-	-	-	-	-	1.644317	2.662633
TSa	20.5257	-	-	-	-	-	-	-	1.018317
GaTSa+BP	19.50738	-	-	-	-	-	-	-	-

The Friedman test result for time series prediction was equal to 3.32143 which is greater than critical level (2.98), so the null hypothesis was rejected. The Nemenyi post-hoc test was performed and the results are shown in Table 10. The significant differences can be seen in bold in the table. This result is based on MSD equal to 0.039686.

**Table 10:** Nemenyi test for the time series prediction problem.

Algorithm		KAT	BatRM-S	T-Logistic BatDNN	gHFSNN (triangular)	gHFSPNN (Gaussian)	gFSPNNT (triangular)	gFSPNNT (Gaussian)
	Mean	0.13405	0.27685	0.16635	5.77575	5.1381	5.659	5.17205
KAT	0.13405	-	**0.1428**	0.0323	**5.6417**	**5.00405**	**5.52495**	**30.82795**
BatRM-S	0.27685	-	**-**	**0.1105**	**5.4989**	**4.86125**	**5.38215**	**4.8952**
T-Logistic BatDNN	0.16635	-	-	-	**5.6094**	**4.97175**	**5.49265**	**5.0057**
gHFSNN (triangular)	5.77575	-	-	-	-	**0.63765**	**0.11675**	**0.6037**
gHFSPNN (Gaussian)	5.1381	-	-	-	-	-	**0.5209**	0.03395
gFSPNNT (triangular)	5.659	-	-	-	-	-	-	**0.48695**
gFSPNNT (Gaussian)	5.17205	-	-	-	-	-	-	-

### Results for a real-world water quality prediction problem

The final part of this study examined the performance of the KA and KAT when applied to real-world water quality data. This experiment is conducted on multivariate time series data taken from a weather station near Kajang in the province of Selangor in Malaysia. The data consists of monthly water quality data reports for the years 2004 to 2013. In this study the data is used as a prediction problem. The data has 13 attributes: SFLOW, TEMP (degrees C), TUR (NTU), DS (mg/l), TS (mg/l), NO_3_ (mg/l), PO_4_ (mg/l), DO (mg/l), BOD (mg/l), COD (mg/l), SS (mg/l), pH (unit), and NH_3_-NL (mg/l). All 13 attributes are used as inputs for the ANN and the last six attributes listed above are presented as the outputs of the ANN. These six attributes are considered as outputs because they are the most critical attributes for water quality prediction. Seventy per cent of the data is used as a training set and 30% is employed as a testing set. The data is normalized into the range of [0, 1] by using the min-max normalization technique. To validate the result a 10-fold cross-validation technique was used. A one-step ahead prediction is carried out. The average of 30 runs for the prediction are shown in Table 11. It can be seen from the table that the KAT performs better than the KA due to the inclusion of the treatment process in the former.

**Table 11:** Prediction results of KA and KAT for real-world water quality data.

Feature	Criteria	KAT	KA
NH_3_-NL	Training error	**0.3404**	0.3748
	Testing error	**0.3383**	0.3757
PH	Training error	**0.2046**	0.2967
	Testing error	**0.209**	0.3164
SS	Training error	**0.1745**	0.2781
	Testing error	**0.1758**	0.2793
COD	Training error	**0.4081**	0.4128
	Testing error	**0.4159**	0.4187
BOD	Training error	**0.2046**	0.2485
	Testing error	**0.2048**	0.2707
DO	Training error	**0.2153**	0.2585
	Testing error	**0.2569**	0.2607

The *p-*values for KA and KAT are calculated to ascertain whether there is a significant difference between the results of KA and KAT. Table 12 shows the results of this test. The critical value is 0.05. From the table it can be observed that all the *p*-values are much lower than the critical value. This superior result is due to the treatment process embedded in the KAT.

**Table 12:** Pairwise comparison of *p-*values for real-world water quality data.

Feature	KA	
	Training error	Testing error
NH_3_-NL	**2.01E-27**	**4.49E-24**
PH	**9.87E-40**	**4.49E-24**
SS	**9.87E-40**	**3.44E-37**
COD	**2.65E-06**	**1.27E-02**
BOD	**1.88E-30**	**4.44E-31**
DO	**2.98E-30**	**1.73E-03**

## Conclusion

This paper presented a modification of the KA, which is a recently proposed optimization algorithm inspired by the functionality of the kidneys in the human body. The modification, coded as KAT, was a simulation of the treatments that would be required if the functionality of the kidneys in human body were reduced. The modification relied on a calculation of the GFR, where a GFR greater than 60 means that functionality of the kidneys is normal, a GFR between 15 and 60 means that treatment is needed to improve the functionality of the kidneys (which equates to lifestyle changes or medication in the real world), and a GFR of less than 15 means that kidney failure has occurred and a treatment is needed to replace the original kidney function (which equates to dialysis in the real world). This concept was used to simulate the type of treatment to be added to KA when the KA functionality was reduced and the GFR was less than 60. The GFR formulas for minimization and maximization were given. This modification provided the algorithm with more exploration. This enabled it to achieve better results when it was applied to test functions and to benchmark classification and time series prediction problems. The proposed method was also applied to real-world water quality prediction. The statistical analysis of all these applications showed that the proposed method had ability to improve the optimization outcome.

In this work the process of KA is used again when kidney fails and dialysis using dialysis machine is needed. The process of dialysis machine can be simulated in details for the kidney failure as future work of this study.

## Supporting information

S1 Fig Flowchart of KAT. (DOCX)
