# Supplementary figures and images for: Kidney-inspired algorithm with reduced functionality treatment for classification and time series prediction
Source: PLoS One. 2019 Jan 4;14(1):e0208308. doi: 10.1371/journal.pone.0208308 (PMC6319704; doi:10.1371/journal.pone.0208308)

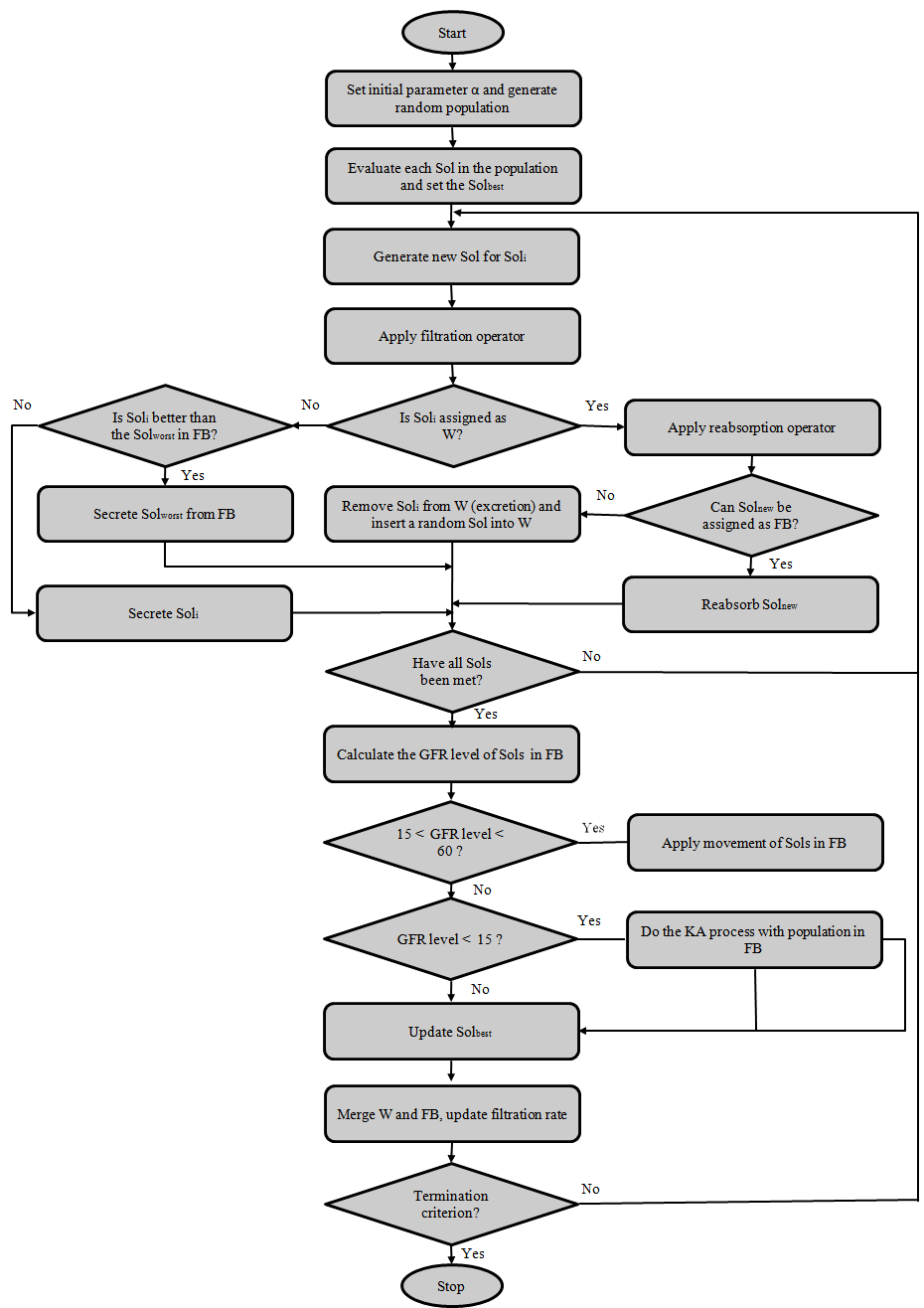

Supplement: S1 Fig — (DOCX) [file pone.0208308.s001.docx]
